# Inversion of the Uterus

**Published:** 1899-07-22

**Authors:** 


### INVERSION OF THE UTERUS.

Dr. Hellier[1], of Leeds, relates a case of chronic inversion of the uterus which was reduced by the use of Aveling's repositor. He remarks that considering the rarity of acute inversion, and that for the condition to become chronic it must remain unreduced, and the patient must survive the danger of a speedy and fatal termination which this involves, such a thing as chronic inversion of the uterus must be excessively rarely met with. In the case related the woman, who was 24 years of age, was admitted on April 11th complaining of flooding, having been confined of her second child the previous October. Five days after labour she had bearing down pains and a tumour presented at the vulva. On the medical attendant being summoned he found inversion of the uterus, which he reduced without chloroform and without difficulty. After the reduction the patient had no more pain and no further examination was made. At the end of three months from that time she had four days' normal menstruation, and at the end of the fourth month she had a week of excessive loss. The next month she began to flood, and had continued to lose profusely till her admission to hospital. On April 17th an attempt was made to reduce the inversion by taxis, but failed, and on April 25th Aveling's repositor was applied in accordance with Aveling's directions. The cup had a diameter of one and a-half inches, and it was kept in position without any packing. On April 27th, after fifty-one hours of application, the fundus was found to have gone up, the cup of the repositor having gone up with it and being firmly grasped by the cervix. To remove the repositor ether had to be administered. It was then found that there was still partial inversion of the fundus, which felt exactly like a polypus within the os internum. On making steady digital pressure on this tumour and counter extension on the cervix, by means of three pairs of vulsella, reduction was easily completed. The cavity of the uterus was washed out and was packed with iodoform gauze. The history of this case bears out the statement that if a case of inversion survives the stage of involution it may give but little trouble till menstruation is re-established, when it causes danger by way of hæmorrhage.

[1]Lancet, July 15.

